# Occurrence and evolution of cannibal behaviour in extant snakes

**DOI:** 10.1111/brv.70097

**Published:** 2025-11-02

**Authors:** Bruna B. Falcão, Vinícius A. São Pedro, Omar M. Entiauspe‐Neto

**Affiliations:** ^1^ Universidade Federal de São Carlos, Laboratório de Estudos Zoológicos do Alto Paranapanema (LEZPA) campus Lagoa do Sino, Rodovia Lauri Simões de Barros, Km 12 SP‐189 18290‐000 Buri SP Brazil; ^2^ Programa de Pós‐Graduação em Recursos Florestais, Departamento de Recursos Florestais Escola Superior de Agricultura ‘Luiz de Queiroz’, Universidade de São Paulo Av. Pádua Dias 11 13418‐900 Piracicaba SP Brazil; ^3^ Laboratório de Coleções Zoológicas Instituto Butantan 05503‐90, Av. Vital Brazil, 1500, Butantã São Paulo SP Brazil; ^4^ Programa de Pós‐Graduação em Zoologia, Departamento de Zoologia Instituto de Biociências, Universidade de São Paulo Rua do Matão, 321, Travessa 14 05508‐090 São Paulo SP Brazil

**Keywords:** serpents, feeding behaviour, ophiophagy, predator–prey interaction, conspecific predation

## Abstract

Extant snakes (Serpentes) are a highly diverse group of squamate reptiles, which have independently evolved key morphological adaptations to consume a large variety of vertebrate and invertebrate prey. While these predator–prey interactions have been widely addressed by several studies, little is known regarding the occurrence of cannibal behaviour in snakes, with scattered reports restricted mostly to natural history notes or incidental records. Here we provide an extensive review of cannibalism in extant snakes, with 503 case events available from the literature, that encompass at least 207 species in 15 families, in both captivity and the wild. For all case events, including those with and without location data, cannibal incidents occurred mostly within the Colubridae (29.0%), Viperidae (21.2%), and Elapidae (18.9%). As cannibalism in snakes has been hypothesized to be a random event, we test whether theories of foraging and feeding strategies apply to cannibal behaviour, finding that prey and predator body size are positively correlated (*R* = 0.81). Furthermore, we explore the evolution of cannibal behaviour in extant snakes, inferring a maximum‐likelihood ancestral character estimation of cannibalism over a family‐level phylogenetic tree, which revealed independent evolution of this behaviour at least 11 times during the evolutionary history of snakes. Cannibalism also appears to be correlated with macrostomate mandibular morphotypes, occurring only in Alethinophidia, and being absent in most snakes that possess mandibular morphotypes with reduced mobility. We conclude that cannibal behaviour appears to be widespread in extant snakes, and possibly represents an opportunistic behaviour that can be related to their evolutionary history, dietary and morphological specialization, environment, and other ecological correlates.

## I. INTRODUCTION

Cannibalism can be characterized as a predatory event where an individual consumes another from its own species. It is a common and taxonomically widespread behavioural trait (Fox, 1975; Polis, 1981; Smith & Reay, 1991; Fouilloux, Ringler & Rojas, 2019; Bose, 2022). Consuming a conspecific might seem a counterproductive ecological behaviour, and was considered a maladaptive trait or even a stress‐induced laboratory artefact by early researchers (Hauschka, 1952; Chardine & Morris, 1983). Since then, a variety of contexts and adaptive hypotheses have been proposed to explain the occurrence of cannibal behaviour, including provision of energetic benefits (Polis & Myers, 1985; Lourdais *et al*., 2005), control of brood size, reuse of energy invested in non‐viable offspring during parental care (Lourdais *et al*., 2005; Bose, 2022), response to low resource availability (Coelho‐Lima, Cardoso & Passos, 2021), decreased competition for offspring (Maia & Travaglia‐Cardoso, 2017), sexual cannibalism (Rivas & Owens, 2000), population control (Paterna, 2023), and opportunistic predation (Maritz, Alexander & Maritz, 2019). Cannibal behaviour has been reported in most taxa, including arthropods, fish, birds, mammals, amphibians, and reptiles (Bose, 2022). While common and widely evaluated in taxa such as bony fishes (Bose, 2022), honey bees (Schmickl & Crailsheim, 2001) and primates (Goodall, 1977; Culot *et al*., 2011), little is known regarding its occurrence in solitary predators such as snakes.

Extant snakes (Serpentes, Linnaeus 1758) represent a highly successful clade of carnivorous tetrapods, with approximately 4145 species. Snakes are found on all continents except Antarctica, and have adapted to most available ecological niches (Vitt & Caldwell, 2014; Uetz *et al*., 2024). Despite being elongated and limbless, snakes are efficient predators that have developed a wide array of morphological specializations, enabling them to feed on diverse groups of vertebrates (amphibians, fish, reptiles, birds, mammals) and invertebrates (crustaceans, annelids, molluscs, arthropods) (Savitzky, 1983; Greene, 1997; Cundall & Greene, 2000; Oliveira *et al*., 2023). These morphological adaptations include the early evolutionary development of a venom delivery system, with a venom gland, associated muscles and canaliculate maxillary teeth that enable chemical incapacitation of prey, and cranial modifications for hyperkinesis that allow an enhanced mouth gape for the consumption of larger prey (Jackson, Kely & Brainerd, 2004; Jackson, Jouanne & Vidal, 2019; Zaher *et al*., 2019, 2023). Some snake clades have developed markedly specialized diets, as seen in ophiophagous species of *Boiruna* Zaher, 1996, *Clelia* Fitzinger, 1826, *Lampropeltis* Fitzinger, 1843, *Micrurus* Wagler, 1824, and *Ophiophagus* Günther, 1864, which have non‐conspecific snakes as major or exclusive components of their diets (Roze, 1996; Greene, 1997; Jackson *et al*., 2004; Gaiarsa, de Alencar & Martins, 2013; Silva‐Jr., 2016). The intriguing phenomenon of cannibalism, a special case of ophiophagy, has been reported in several snake taxa. Although no dietary studies have yet assessed the proportion of the diet comprising conspecifics for snakes, scattered reports of cannibalism remain pervasive for most families. Despite seemingly being a widespread ecological interaction, no comprehensive review of cannibal behaviour exists for snakes to date, with most reports available only in the form of short communications or incidental records, often published in grey literature.

Here we provide the first synthesis of cannibalism for extant snakes, with an extensive review of published records for this behaviour. We collated available information on taxonomy, ontogeny, ecology, morphometry, observation setting, geographic location and publication type, in order to evaluate trends in reported cannibal behaviour. We test whether theory on foraging and feeding strategies regarding the prey–predator size relationship in snakes (Arnold, 1993; Schoener, 1971) applies to cannibal behaviour, using a regression of prey on predator body size as a proxy to infer a size effect in this predation dynamic. These hypotheses predict that predators will select larger prey individuals when prey are not scarce, to optimize their energetic return. Furthermore, we explore whether evolutionary dynamics have shaped the occurrence of cannibalism in snakes, evaluating its distribution with an ancestral character estimation over a family‐level phylogeny of extant snakes. We also compare the occurrence of cannibalism with the presence of different mandibular morphotypes, to investigate its relationship with hyperkinesis. We discuss patterns and trends that may be associated with cannibalism in snakes, evaluating possible causes or triggers for the occurrence of this behaviour in the wild and captivity.

## II. MATERIAL AND METHODS

### (1) Literature searches and data compilation

We conducted systematic searches on *Web of Science*, *Google Scholar*, and *Scopus* databases (last accessed 03 January 2025) to identify studies reporting cannibal behaviour in snakes. We used the following Boolean search string: ‘cannibalism’ AND ‘snakes’ OR ‘cobras’ OR ‘serpentes’ OR ‘Acrochordidae’ OR ‘Aniliidae’ OR ‘Anomalepididae’ OR ‘Atractaspididae’ OR ‘Boidae’ OR ‘Bolyeriidae’ OR ‘Calabariidae’ OR ‘Calamariidae’ OR ‘Candoiidae’ OR ‘Charinidae’ OR ‘Colubridae’ OR ‘Cyclocoridae’ OR ‘Cylindrophiidae’ OR ‘Dipsadidae’ OR ‘Elapidae’ OR ‘Erycidae’ OR ‘Gerrhopilidae’ OR ‘Grayiidae’ OR ‘Homalopsidae’ OR ‘Lamprophiidae’ OR ‘Leptotyphlopidae’ OR ‘Loxocemidae’ OR ‘Natricidae’ OR ‘Pareidae’ OR ‘Psammophiidae’ OR ‘Pseudoxenodontidae’ OR ‘Pseudoxyrhophiidae’ OR ‘Pythonidae’ OR ‘Sanziniidae’ OR ‘Sibynophiidae’ OR ‘Tropidophiidae’ OR ‘Ungaliophiidae’ OR ‘Uropeltidae’ OR ‘Viperidae’ OR ‘Xenodermidae’ OR ‘Xenopeltidae’ OR ‘ophiophagy’ NOT ‘lizards’. We combined Portuguese and English search terms to increase coverage. The raw list of potentially relevant studies was then individually verified for the presence or absence of recorded events. We also checked individual references contained in the retained articles for potentially relevant works.

We compiled a total of 299 papers regarding cannibalism in snakes. For each included paper, we collected the following information, where possible: (*i*) taxonomic information (family; genus; species name as referred to in the study; current taxonomic species name); (*ii*) ecological habits (recorded food habits; ophiophagy records); (*iii*) predator age, size and sex; (*iv*) prey age, size and sex; (*v*) event type; (*vi*) ingestion direction (head first; tail first); (*vii*) occurrence type; (*viii*) year; (*ix*) geographic location; (*x*) publication type. It should be noted that ophiophagy records in the diet of a species do not necessarily imply a snake‐specialist diet; as comprehensive dietary studies were not available for the majority of evaluated species, these two terms are treated separately here.

For prey and predator individual data, we collected information on age, sex, and size. Age was determined as: (*i*) undeveloped eggs; (*ii*) eggs; (*iii*) newborn individuals; (*iv*) stillborn individuals; (*v*) juveniles; (*vi*) adults. Where available, size was recorded as snout–vent length (SVL, measured from the tip of rostrum to cloacal opening), or total length (TTL = SVL + tail length).

We classified event type as: (*i*) maternal–offspring cannibalism (MC), when a female parent deliberately ingests its offspring at any ontogenetic stage; (*ii*) cannibalism between offspring (CBO), when offspring ingest each other; (*iii*) sexual cannibalism (SC), when cannibalism takes place in mature individuals of opposite sexes during a mating event or season; (*iv*) combat‐dance cannibalism (CDC), when males engage in cannibalism after a combat‐dance mating display; (*v*) undetermined (UND), when no other reason could be ascertained and the event is likely opportunistic.

For occurrence type, we classified events as: (*i*) wild (presumably natural) events, when a cannibal event was observed in a natural setting, and individuals were observed or inferred to be engaging in cannibalism under natural circumstances; (*ii*) captivity, when a cannibal event took place in captivity; (*iii*) dissection/undetermined, when a cannibal event was discovered by dissecting a specimen, and its circumstances were unknown.

We determined publication type as: (*i*) full‐length articles, for publications which contained three or more sections; (*ii*) short communications, for publications up to five printed pages, with fewer than three sections; (*iii*) other types of published material, for publications within books, magazines, dissertations, or which did not fit publication‐type criteria *i* or *ii*.

### (2) Event data analysis

For the compiled works, we generated a raw data set of available information on snake cannibal events (see online Supporting Information, Appendix S1). From this data set, we used percentages to summarize the proportion and predominance of events per higher‐level taxonomic group, occurrence type, event type, and ecological data recorded for each species. We also investigated whether theories of feeding strategies and foraging (Schoener, 1971; Arnold, 1993) apply to cannibal behaviour by investigating the relationship between prey and predator body size. For our analyses, we employ data from records obtained in natural settings, in captivity, and in unknown conditions. Considering the absence of precise collection‐locality data for captive specimens, these were included in their country of captivity, rather than origin. Records without locality data at country level were not included in our geographic analyses. We generated a data set (Appendix S2) with log‐transformed TTL for prey and predators to perform a regression to test for a size effect in cannibal behaviour. First, we checked that prey and predator log‐transformed TTL/SVL followed the assumptions of a normal distribution with a Shapiro–Wilk test, and then used a linear regression and calculated Pearson correlation coefficients. If cannibalism represents a random or maladaptive behaviour, we would expect no correlation between predator and prey size. We also performed exploratory analyses between snake families using boxplots. We explored patterns of reporting of cannibal behaviour by separating the data according to publication year, and analysed these using an empirical cumulative distribution function in R. Unless otherwise stated, *N* = number of cannibalism records. All analyses were conducted in R (R Core Team, 2025) and all scripts used are available at: https://github.com/omarentiauspe/cannibalism_snakes.

### (3) Comparative phylogenetic analyses

We evaluated the evolution of cannibal behaviour in extant snakes using the phylogenetic tree of Burbrink *et al*. (2020) as a framework. We pruned a phylogenetic tree inferred by these authors (dated species tree for Squamata) within the clade Serpentes into a family‐level Newick‐format phylogeny, with 37 extant snake families as tips. In order to understand state character changes within the evolutionary history of snake families, we considered cannibalism as a binary discrete character, coded as ‘present’ or ‘absent’ for each tip based on verified occurrences (Appendix S1). We employed an ancestral character estimation (ACE) analysis to estimate the probability of cannibal behaviour at each internal node (i.e. common ancestor) using the ‘ancr’ function within the *Phytools* 2.0 package (Revell, 2024), which computes empirical Bayesian posterior probabilities as marginal ancestral states conditioned by fitted models on a set of observed (tip states) information. We fitted our data with individual Markov K (Mk) models, a stochastic model family that describes a continuous time Markov chain with *k* possible states for discrete data (Revell, 2014; Revell & Harmon, 2022). We evaluated the following Mk models: Symmetric (SYM), for symmetric backward and forward rates for all permitted transitions; All Rates Different (ARD), where all rates are different for permitted transitions; and Equal Rates (ER), for equal rates for all permitted transitions. The Mk models were tested for statistical congruence with log‐likelihood (LnL), Akaike Information Criterion (AIC), and weighted Akaike Information Criterion (AICw) values, extracted using built‐in R software functions. We arbitrarily define a threshold of ≤90% reconstructed node likelihood for considering a character to be gained or lost. In order to evaluate the percentage of cannibal snakes that also prey upon snakes in the wild (natural ophiophagy), we mapped the relative percentage of natural ophiophagy among cannibal species per family, and inferred these percentages as a numerical vector of phenotypic trait values for each species, using continuous character mapping analysis with the ‘contmap’ function within the *Phytools* 2.0 package (Revell, 2024).

Snakes historically have been classified into two groups regarding their mandibular morphotypes: macrostomates, which have cranial modifications for hyperkinesis that allow them to consume larger prey, and microstomates, which have a reduced mouth gape that is considered to be symplesiomorphic with non‐ophidian lizards. While once thought to represent two monophyletic groups, it has been shown that macrostomy and microstomy morphotypes likely evolved independently more than once in snakes, with a paraphyletic microstomate Scolecophidia (Strong, Scherz & Caldwell, 2021), and the first records of macrostomy appearing in the common ancestor of basal snakes, such as pachyophiids, Cenozoic Australian ‘madtsoiids’ and alethinophidians during the Cretaceous (Zaher *et al*., 2023). In order to evaluate the influence of mandibular morphotypes in the occurrence of cannibalism, we evaluated the occurrence of this behaviour in microstomate scolecophidian and macrostomate alethinophidian extant snakes (see Miralles *et al*., 2018), and we also test the classification proposed by Strong *et al*. (2021), where ‘snout‐shifting’, ‘axle‐brace maxillary raking’, ‘mandibular raking’, and ‘single‐axle maxillary raking’ are morphotypes of early‐diverging alethinophidians, anomalepidids, leptotyphlopids, and typhlopoids with reduced mandibular mobility, while alethinophidians with ‘caenophidian‐type macrostomy’ and ‘booid‐type macrostomy’ have enhanced jaw hyperkinesis. However, it should be noted that Strong *et al*. (2021) reconstructed ‘snout‐shifting’ as a reduced jaw kinesis morphotype for Amerophidia without sampling Tropidophiidae, to which the latter is tentatively assigned here despite showing significant osteological differences from its counterpart Aniliidae (see Rieppel, 2012; Ortega‐Andrade *et al*., 2022); ‘snout‐shifting’ is herein considered as a macrostomate phenotype. We mapped mandibular morphotypes and cannibalism occurrence as discrete characters onto the tips of the same phylogeny referred to above, and visually inspected the concordance of these characters as an exploratory analysis. All analyses were conducted in the R Software Environment (R Core Team, 2025).

## III. RESULTS

**Fig. 1:**
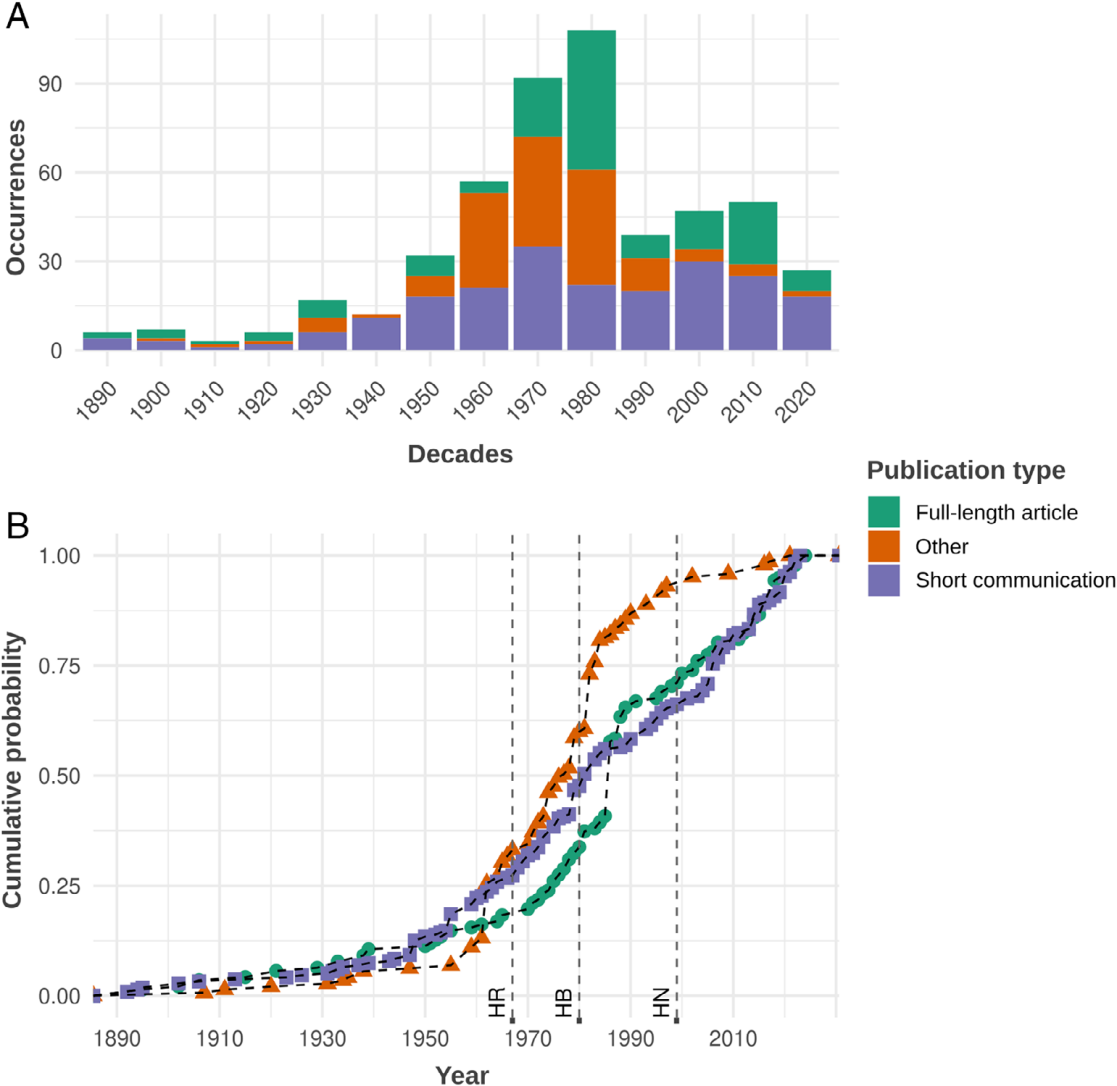
Reporting patterns for cannibalism events in extant snakes. (A) Published occurrences per decade. (B) Cumulative distribution per year and publication type. Establishment dates of major journals that publish short communications are indicated with dashed lines and circles: HR, *Herpetological Review*; HB, *Herpetological Bulletin*; HN, *Herpetological Notes*.

**Fig. 2:**
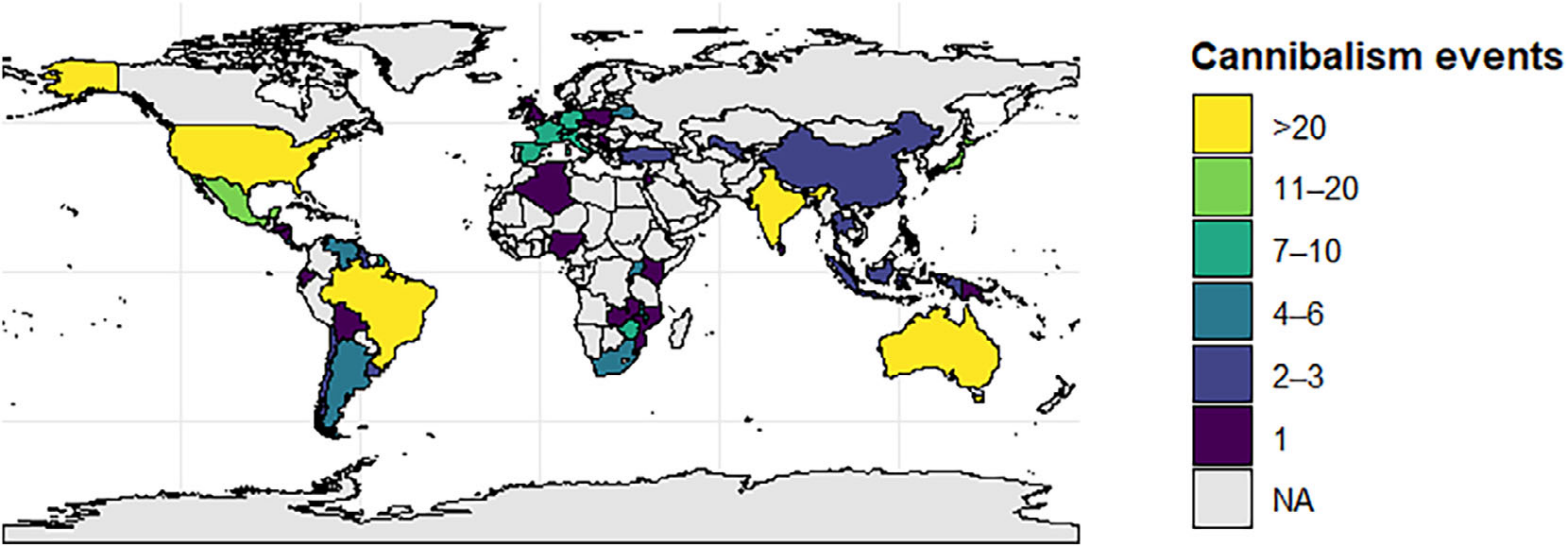
Global geographic distribution of cannibalism events reported in snakes. Heat map showing the number of cannibalism events reported per country. Countries with no data (NA) are shown in grey. Events from Wales were grouped under UK.

**Fig. 3:**
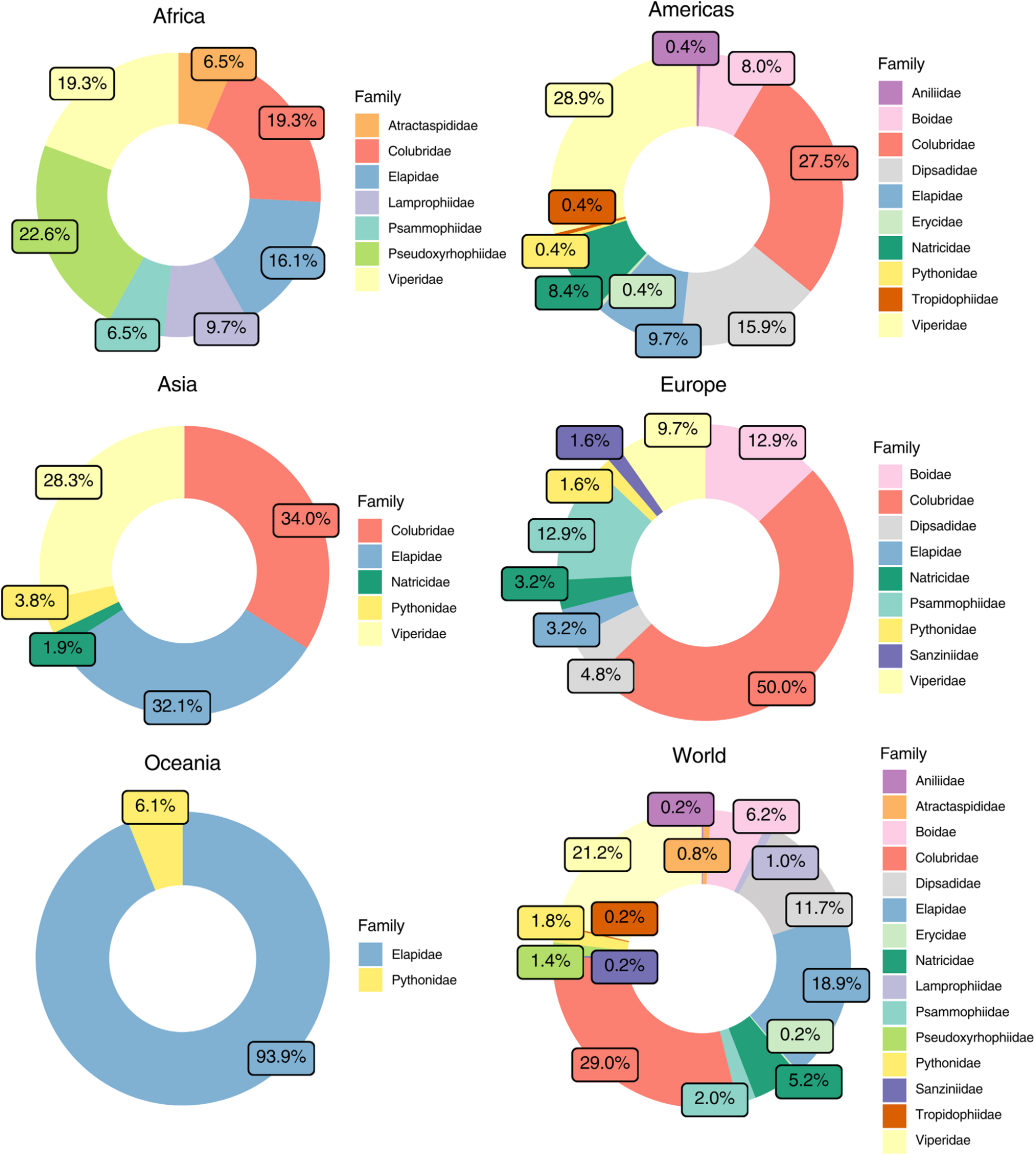
Geographic distribution of reported cannibalism events per snake family on individual continents and worldwide, for which locality data were available. Total sample sizes: Africa (*N* = 31), Americas (*N* = 226), Asia (*N* = 53), Europe (*N* = 62), Oceania (*N* = 34), on all continents: 406.

We identified a total of 503 cannibalism events reported in snakes, distributed in 299 published works (Appendix S1). The majority of these cases were published as short communications (*N* = 216; 42.9%), followed by other types of published material (*N* = 145; 28.8%) and full‐length articles (*N* = 142; 28.2%). We found no evidence of marked temporal trends between publication types, although there has been an increase in reports as ‘other types of published material’, with a higher cumulative probability from 1960 onwards (Fig. 1B). The earliest reports of cannibalism date from 1892, and the highest numbers of publications appeared in the 1980s (*N* = 104), 1970s (*N* = 91), and 1960s (*N* = 57) (Fig. 1A). We located records for 55 different countries (Fig. 2), on all continents where snakes are present. The families in which cannibalism was recorded most frequently varied by continent: Pseudoxyrhophiidae had the majority of cannibal records in Africa (*N* = 7 records; 22.6% of records from Africa), Viperidae in the Americas (*N* = 68 records; 28.9%), Colubridae in Asia (*N* = 18 records; 34.0%) and Europe (*N* = 31 records; 50.0%), and Elapidae in Oceania (*N* = 32 records; 93.9%) (Fig. 3). The most records originated from the USA (*N* = 144; 35.5%), Australia (*N* = 33; 8.1%), Brazil (*N* = 25; 6.2%) and India (*N* = 24; 5.9%) (Fig. 2).

Cannibalism was recorded for a total of 207 snake species (Appendix S2, Sheet S4), distributed in 15 families: Aniliidae (one species; *N* = 1; 0.2% of all records), Atractaspididae (four species; *N* = 4; 0.8%), Boidae (11 species; *N* = 31; 6.2%), Colubridae (42 species; *N* = 146; 29.0%), Dipsadidae (32 species; *N* = 59; 11.7%), Elapidae (46 species; *N* = 95; 18.9%), Erycidae (one species; *N* = 1; 0.2%), Lamprophiidae (three species; *N* = 5; 1.0%), Natricidae (nine species; *N* = 26; 5.2%), Psammophiidae (three species; *N* = 10; 2.0%), Pseudoxyrhophiidae (one species; *N* = 7; 1.4%), Pythonidae (six species; *N* = 9; 1.8%), Sanziniidae (one species; *N* = 1; 0.2%), Tropidophiidae (one species; *N* = 1; 0.2%) and Viperidae (46 species; *N* = 107; 21.2%) (Fig. 3).

**Fig. 4:**
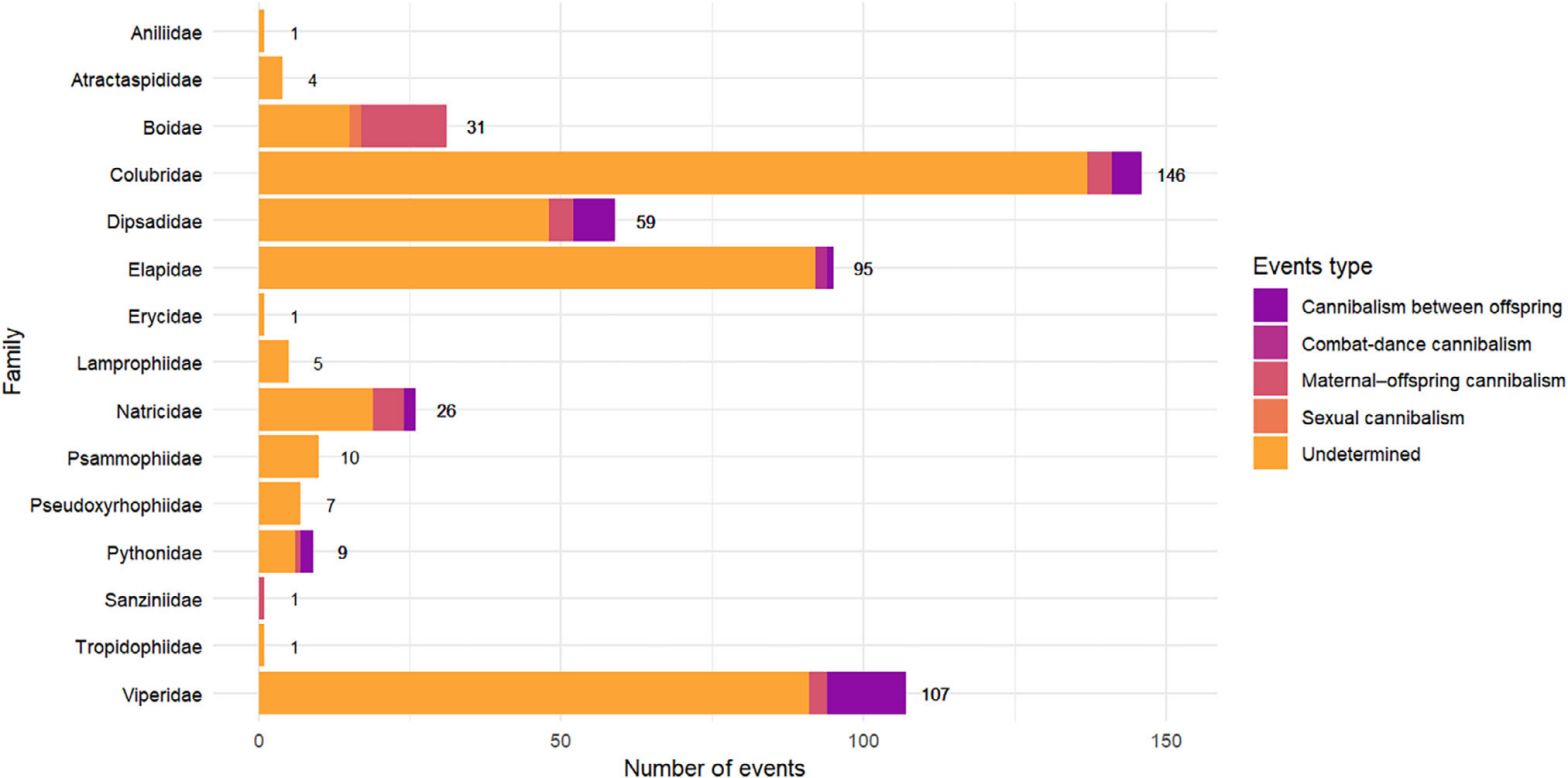
Cannibalism events by snake family and event type.

Most cannibalism records were considered to be opportunistic, with ‘undetermined’ (i.e. opportunistic) events representing 86.9% (*N* = 437) of cases and present in all families for which cannibalism was recorded. Maternal–offspring cannibalism was identified in 6.4% (*N* = 32) of cases, of which most were in Boidae (*N* = 14; 43.8%), followed by Natricidae (*N* = 5; 15.6%), Colubridae (*N* = 4; 12.5%), Dipsadidae (*N* = 4; 12.5%), Viperidae (*N* = 3; 9.4%), Pythonidae (*N* = 1; 3.1%) and Sanziniidae (*N* = 1; 3.1%) (Fig. 4).

Sexual cannibalism was recorded in two instances for Boidae (0.4%). A total of 30 records (6.0%) were encountered for cannibalism between offspring, with the majority being for Viperidae (*N* = 13; 43.3%), followed by Dipsadidae (*N* = 7; 23.3%), Colubridae (*N* = 5; 16.7%), Natricidae (*N* = 2; 6.7%), Pythonidae (*N* = 2; 6.7%), and Elapidae (*N* = 1; 3.3%). Combat‐dance cannibalism was recorded in two instances for Elapidae (0.4%).

**Fig. 5:**
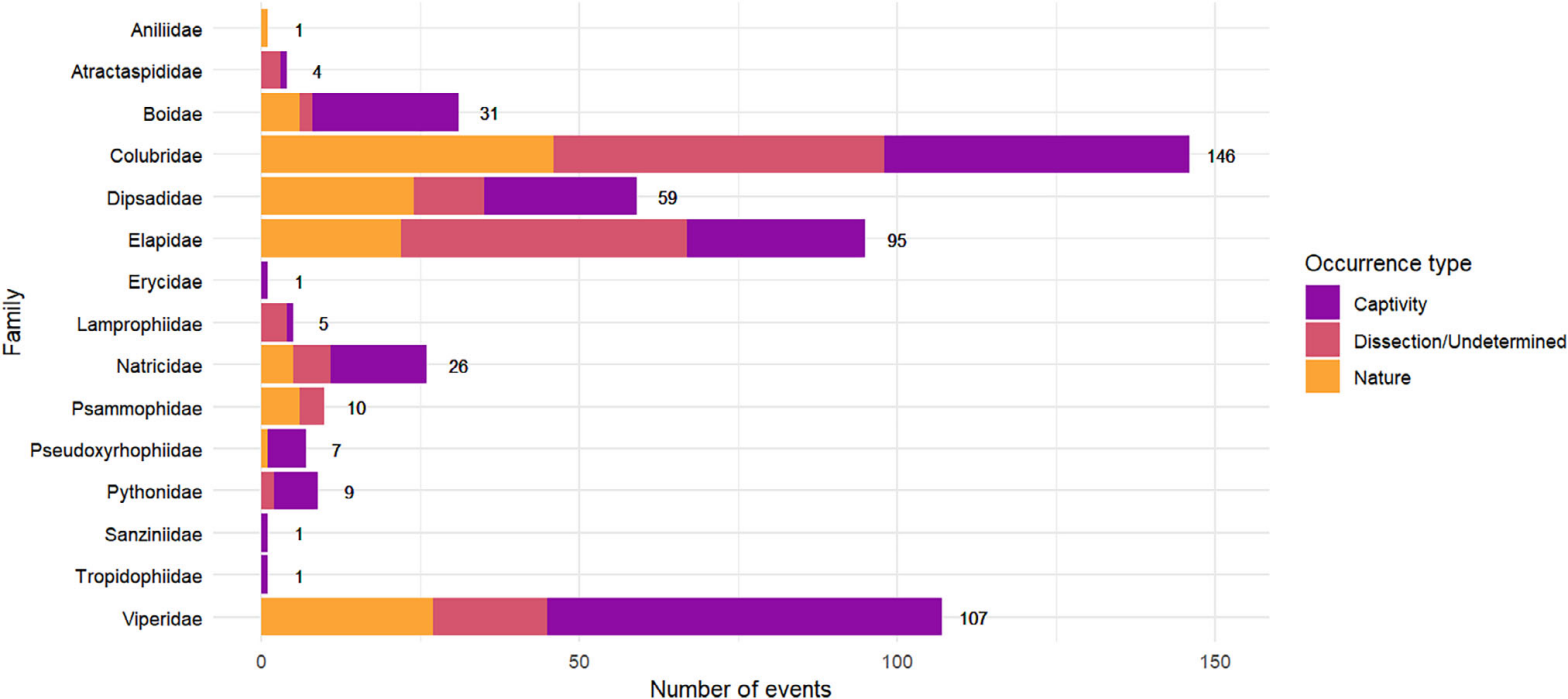
Cannibalism events by snake family and occurrence type.

The majority of cannibalism events were recorded in captivity (*N* = 218; 43.3%), which represented approximately 1.6 times more events than reported from natural settings (*N* = 138; 27.4%). ‘Undetermined’ settings accounted for 147 events (29.3%) (Fig. 5). For most families the majority of records were from captivity, with the highest proportion being in Viperidae (*N* = 62/107; 57.9%), Colubridae (*N* = 48/146; 32.9%), and Elapidae (*N* = 28/95; 29.5%). Only for Aniliidae (*N* = 1/1; 100%) and Psammophiidae (*N* = 6/10; 60.0%) were the majority of records from the wild. While most of the evaluated events did not report ingestion direction (*N* = 432; 85.9%), of those that did (*N =* 71), 83.1% (*N* = 59) reported head‐first ingestion of prey. Almost half of the cannibal snake species have a generalist diet (*N* = 95; 47.7%). Notably, 3.9% (*N* = 8) feed on serpentine or elongated‐bodied animals, and 2.9% (*N* = 6) are strictly ophiophagous (see Appendix S1). Cannibalistic events exclusively observed in nature were documented for 74 species, belonging to nine families and recorded across 41 countries, encompassing all continents. This distribution reflects the global occurrence of cannibalism among snakes and respects the natural biogeographic range of each species and family.

**Fig. 6:**
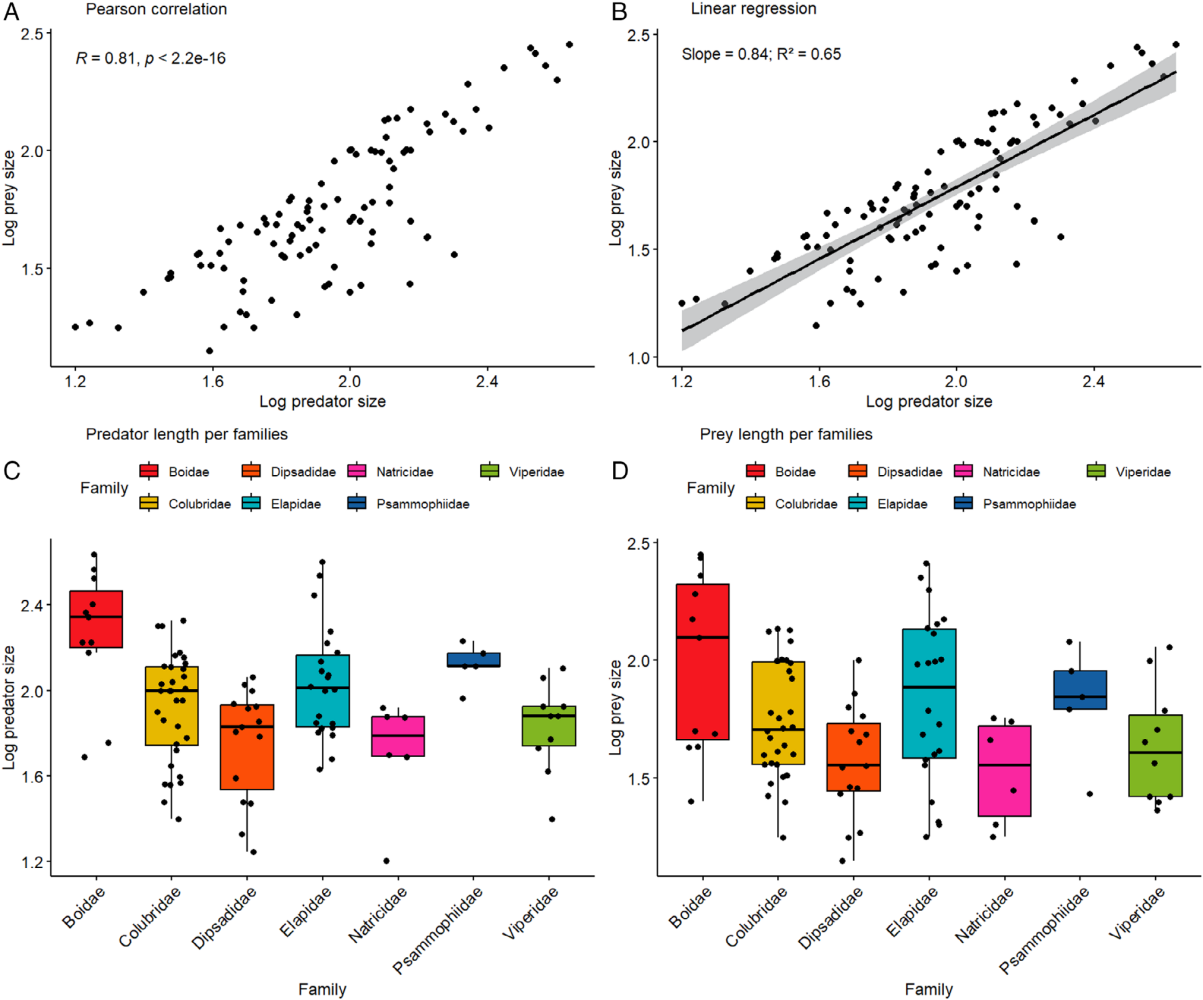
Relationships between log‐transformed total length (TTL)/snout‐vent length (SVL), for predator and prey involved in cannibal events. (A) Pearson correlation between prey and predator size. (B) Linear regression between prey and predator size; shaded region indicates confidence interval (se = TRUE). (C) Boxplots of predator size for families with >5 records of cannibalism. (D) Boxplots of prey size families with >5 records. Boxplot whiskers indicate 25% of maximum and minimum values; horizontal line indicates median value, separating lower and upper quartiles.

We found a positive correlation between log‐transformed predator and prey size (Fig. 6A, B) for 104 events in ten families (Aniliidae, Boidae, Colubridae, Dipsadidae, Elapidae, Lamprophiidae, Natricidae, Psammophiidae, Pythonidae and Viperidae), suggesting that larger snakes may prey upon larger conspecifics. The distribution of predator and prey size was similar among families (Fig. 6C, D).

**Fig. 7:**
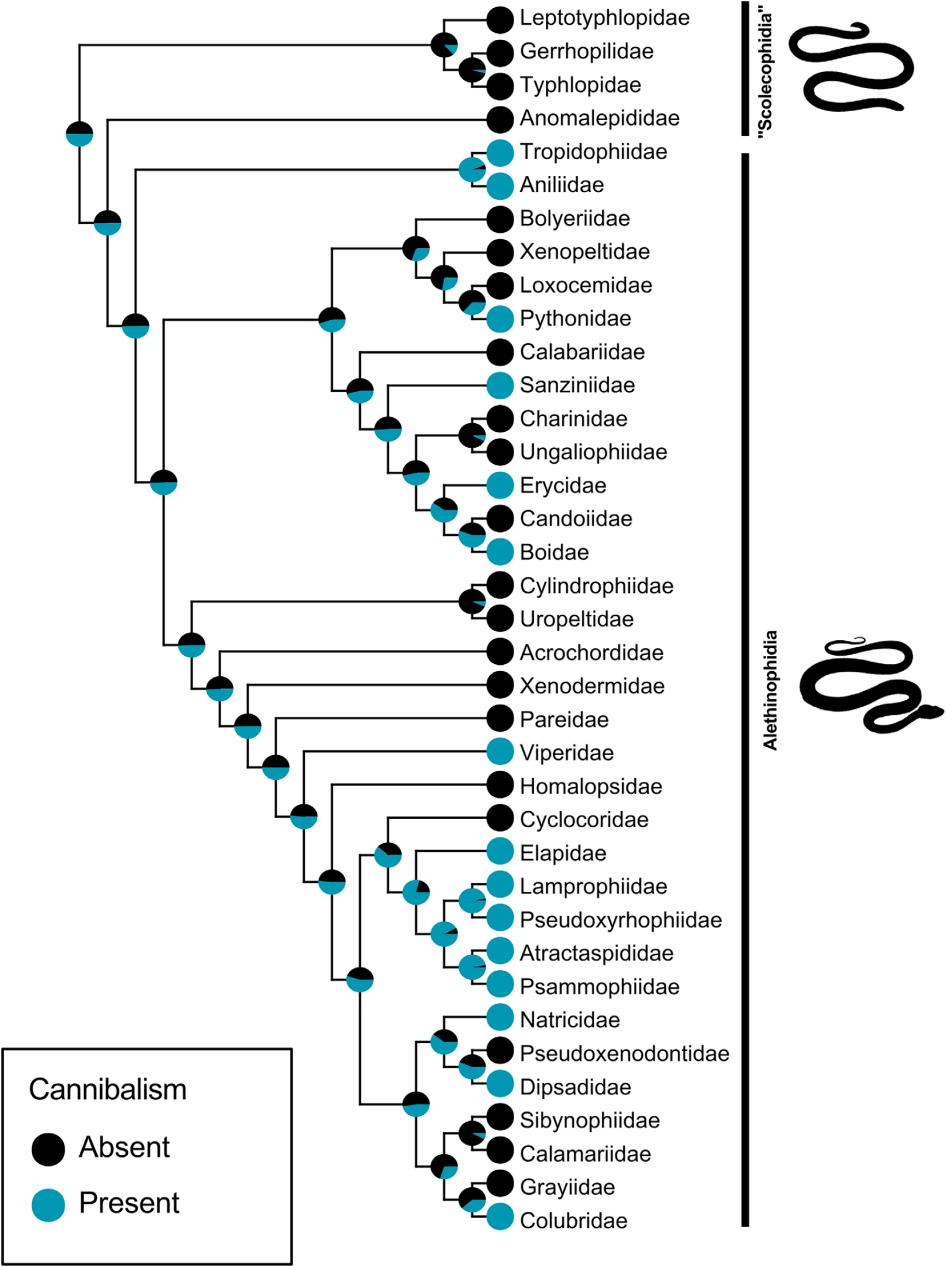
Evolution of cannibal behaviour in extant snake infraorders ‘Scolecophidia’ (paraphyletic blind snakes), and Alethinophidia (monophyletic advanced snakes), as inferred by an Equal Rates (ER) ancestral character estimation model using *Phytools*.

Our phylogenetic analyses recovered identical results with the ER and SYM Mk models (LnL = −24.921; AIC = 51.843; AICw = 0.356), so we selected the ER model for analysis due to its better node resolution for deeper nodes. Our ancestral state reconstruction suggests that cannibalistic behaviour likely appeared at the crown of the extant alethinophidian snakes, recovering a non‐cannibal most recent common ancestor (MRCA) of extant scolecophidian snakes, first appearing at the Amerophidia clade (Aniliidae and Tropidophiidae), with 11 unambiguous independent character gains, and no major suprafamilial taxon character losses (Fig. 7).

**Fig. 8:**
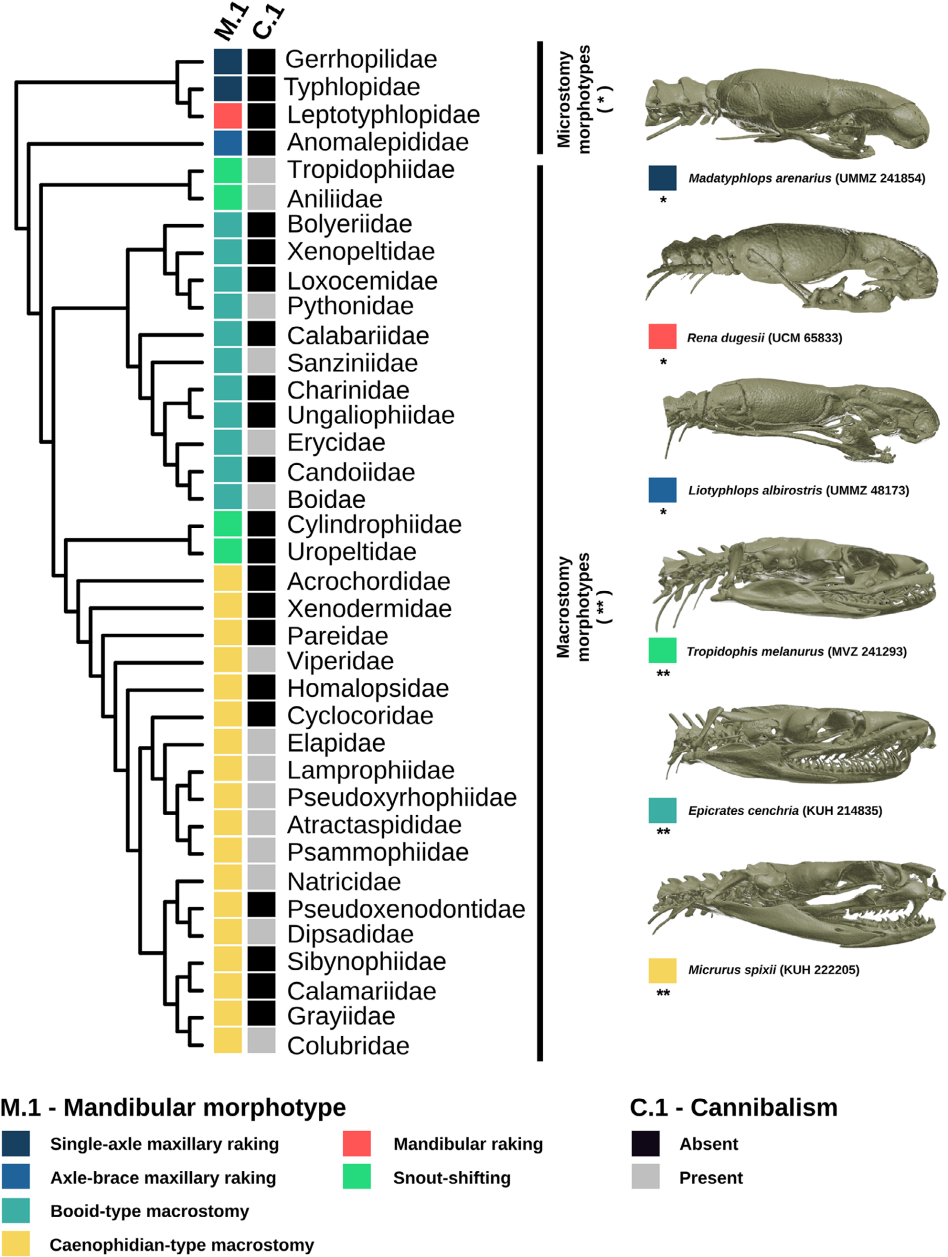
Comparison between evolution of mandibular morphotypes (M.1) modified from Strong *et al*. (2021) and occurrence of cannibalism (C.1) among extant snake families. Inset photographs provide examples of skulls with different mandibular morphotypes. The ‘microstomy morphotypes’ and ‘macrostomy morphotypes’ can be related to the Scolecophidia and Alethinophidia respectively in Fig. 7.

**Fig. 9:**
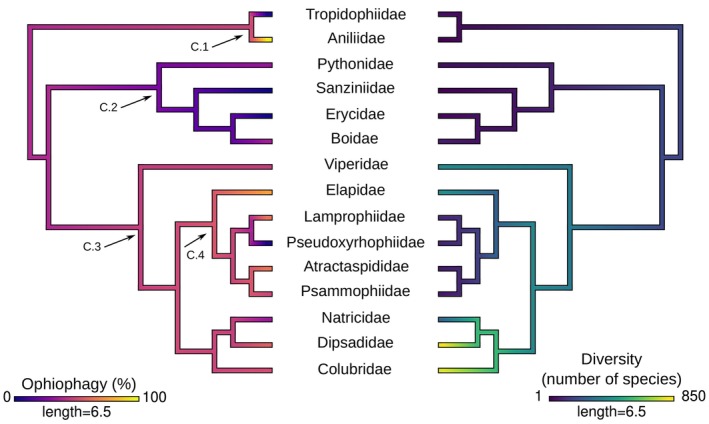
Continuous character mapping for the percentage of ophiophagous behaviour in nature, among species with recorded cannibalism (left), and diversity of species in families with cannibal behaviour (right), inferred using *Phytools*. Diversity of species follows Uetz *et al*. (2024). For this phylogeny, tips for families without recorded cannibal behaviour were removed, and percentage of cannibal taxa that are ophiophagous was calculated. Marked clades of interest are: C.1, Amerophidia; C.2, Constrictores; C.3, Endoglyptodonta; C.4, Elapoidea. [Correction added on 12 December 2025, after first online publication: The legend keys in Figure 9 have been corrected.]

All cannibal species belong to the Alethinophidia, with no records encountered for the paraphyletic scolecophidian species. No cannibalism records were encountered for the single‐axle maxillary raking, axle‐brace maxillary raking, and mandibular raking morphotypes, which are restricted to microstomate scolecophidian snakes. All other macrostomate phenotypes, booid‐type macrostomy, caenophidian‐type macrostomy, and snout‐shifting have records of cannibalism, corroborating that cannibalism may be related to macrostomy and macrophagy (Fig. 8). The Endoglyptodonta clade, which possess a developed venom‐delivery apparatus, has a high proportion of families with cannibal records (9/15), compared to other alethinophidian families (6/18), which might indicate a relationship between venom‐delivery system and cannibalism (Figs 8 and 9; Appendix S2). The Elapoidea clade and Aniliidae display the highest rates of proportional ophiophagy in nature, and cannibalism has been recorded for all families implying a causal relationship between cannibalism and diet rather than an association with species diversity (Fig. 9). Continuous character mapping of the relative percentage of ophiophagy among cannibal species per family revealed a lower rate of natural ophiophagous behaviour for the Constrictores clade (δ 15%), that contains Pythonidae, Sanziniidae, Erycidae and Boidae, and higher rates for Amerophidia and Endoglyptodonta (ε 50%) (Fig. 9).

## IV. DISCUSSION

We present the most comprehensive review of cannibalism in snakes to date, and the first evolutionary analyses of this behaviour for extant snakes. We conclude that cannibalism is a widely distributed behaviour both taxonomically and geographically, has been recorded on all continents in which snakes occur, and in many of the known families. In captivity, confinement in enclosures, movement restriction, lack of enrichment, close proximity to conspecifics, and handling are all known to be stressors (Hediger, 1950; Browning & Veit, 2021) that may trigger cannibalism. In the wild, other factors may be relevant, such as predator abundance, resource availability, and microclimate (Hediger, 1950). Arguably, other influences should also be considered when interpreting our results. For instance, the absence of vipers in Australasia and the evolution of analogous, convergent lineages (e.g. *Acanthophis* Daudin, 1803; Shine, 1980) with a different evolutionary history could perhaps explain the overrepresentation of cannibal elapids in Oceania. The occurrence of pythonid records in the Americas largely derives from captive specimens, as this family was not found naturally on this continent (Vitt & Caldwell, 2014), although invasive pythonid species are now present. We expect a greater number of records for species commonly kept as pets; the majority of exported and imported CITES‐listed snake species are pythonids, colubrids, and elapids (Hierink *et al*., 2020), which were widely represented in our work. Similarly, scientific publications are less likely to originate from regions with developing countries, as exemplified by the low numbers of herpetological publications derived from the Middle East and Africa (Tancoigne *et al*., 2011). Interestingly, the majority (42.9%) of cannibalism records analysed herein were published as short communications, highlighting the importance of this type of publication, as some ecological interactions are only briefly reported and rarely discussed in depth. Indeed, cannibalism in snakes appears to be seldom directly observed in the wild, and when it is, it is usually reported as a short ecological note. We found a reduction in numbers of full‐length articles containing records of cannibalism after the 1980s.

### (1) Cannibalism among snake families and diet

Cannibalism was most frequent in the families Colubridae, Viperidae and Elapidae. For colubrids, in which strictly ophiophagous diets are rare, natural cannibalism events (which represent 31.5% of total events for this family) may be related to stressors, such as the lack of other food resources. Jofré & Reading (2020) suggested that snakes on a commercial pine tree plantation in England may resort to cannibalism when no other prey is available. This behaviour was also recorded by Morais *et al*. (2020) for a dipsadid snake (*Dryophylax phoenix*) in the semiarid Brazilian Caatinga, which might be related to aridity and limited food resources in this environment (Göçmen, Werner & Elbeyli, 2008).

Cannibal events for Viperidae occurred mostly in captivity (*N* = 62/107; 57.9% of events for this family), again likely related to environmental stressors. While evaluating the diet of a viperid species, Powers (1972) suggested that individuals with low food availability in captivity are more likely to express cannibal behaviour when sharing their enclosure with another conspecific. The natural diet of most viperid species sampled in this study largely consists of small mammals or amphibians (Kotler, Blaustein & Dednam, 1993; Martins, Marques & Sazima, 2002; Sant'Anna & Abe, 2007), and no species has been shown to have snakes as a major diet component (Ortiz‐Medina *et al*., 2022). Therefore, it is likely that captivity and its stressors, rather than dietary preferences, is likely to drive cannibal behaviour in vipers.

Elapid snakes are usually ophiophagous (Appendix S2, 78.3% recorded as ophiophagous), that is, usually prey upon other snakes in nature (Maritz *et al*., 2021). Since snake cannibalism is also a type of ophiophagy, the high prevalence of ophiophagous behaviour may explain why elapid snakes have one of the highest rates of cannibalism. It is likely that elapids may not be able to distinguish conspecific from heterospecific prey. Despite having few records of cannibalism behaviour in nature for Atractaspididae and Lamprophiidae, we conjecture that cannibalism may also be common for these groups due to their ophiophagus behaviour, but has been seldom recorded due to their geographic distribution and lack of specimens kept in captivity.

The finding that half of cannibal snake species have a generalist diet corroborates the argument of Hurd (2008) that most generalist predators exhibit cannibal behaviour. However, other factors must be considered. As suggested for an ophiophagous diet, an ovivorous diet may predispose a species to certain types of cannibalism, as seen in *Oligodon taeniolatus* females that ingested their own eggs (Minton & Anderson, 1963). Paterna (2023) suggested that the predation of eggs by other conspecifics might represent a population‐control mechanism, conjecturing that oophagy in this species could be associated with strong territorial behaviour in males of *H. viridiflavus*, although individual data on feeding habits for other species do not seem to indicate any other examples of this behaviour.

### (2) Cannibalism event types

Maternal cannibalism was mostly recorded in Boidae (*N* = 14; 43.8% of recorded maternal–offspring cannibalism among all families, and 45.2% of cannibalism records for this family). Female snakes are known to ingest their own non‐viable offspring selectively (for a review, see Lourdais *et al*., 2005). Eating undeveloped eggs and stillborn neonates may represent maternal care of remaining live offspring by some boid species, which may also incubate their eggs to maintain a stable temperature (Gans, 1996). Removal of non‐viable offspring by ingestion could function to protect viable offspring from disease, as well as removing sources of scent or chemical signals that could attract predators (Gans, 1996; Lourdais *et al*., 2005).

Lourdais *et al*. (2005) showed that maternal cannibalism is also associated with energetic benefits after gestation, assisting the recovery of a boid snake. However, our data suggest that females that eat their viable offspring might express this behaviour due to stressors in captivity. We also found this behaviour at lower frequencies in the families Natricidae, Colubridae, Dipsadidae, Viperidae, Pythonidae, and Sanziniidae when exposed to confined environments in captivity.

Sexual cannibalism, i.e. cannibalism during a mating event or season (Elgar, 1992), was only recorded in a single boid species, *Eunectes murinus* in two separate events (Rivas & Owens, 2000). Rivas & Owens (2000) suggested that sexual cannibalism may represent a strategy for maximizing energy storage, considering that females may spend several months without feeding after mating. Similar behaviour is known in other animal groups, such as arachnids (Elgar, 1992). This behaviour may be facilitated by the marked sexual dimorphism in this species, in which females are considerably larger than males. Glaudas & Fuento (2022) suggested that a low chance of reproduction or refusal to mate by the female might drive male cannibal behaviour. Furthermore, in polyandrous species (e.g. *Eunectes murinus*), an aggregation of multiple males with a single female may reduce the value of each male as a mate, and thus increase its value as prey. This also could represent an adaptive strategy, where preying upon an inferior male will reduce sperm competition (Andrade, 1996). It is therefore possible that sexual cannibalism constitutes a foraging strategy, providing an energetic advantage (Elgar & Schneider, 2004).

The combat‐dance in snakes is an intrasexual conspecific behaviour performed by males seeking to mate with a female during the mating season (Shine, 1978). Combat‐dance cannibalism was recorded in two ophiophagous elapids, *Ophiophagus hannah* (Shankar & Whitaker, 2013) and *Naja nivea* (Maritz, Alexander & Maritz, 2019). No hypotheses have yet been advanced to explain this phenomenon, which may represent a strategy by the winning male to reduce subsequent competition with the losing male, or may simply represent opportunistic predation.

Cannibalism between offspring was seldom recorded (6.0%). Conversely, this behaviour has been widely reported in sharks such as the sand tiger shark *Carcharias taurus*, in which the largest offspring consumes its siblings during gestation (Chapman *et al*., 2013). Such behaviour will increase the fitness of the cannibalistic individual (Roulin & Dreiss, 2012). Although relatively rare, cannibalism between offspring in snakes may be associated with the same drivers as in other animal groups.

### (3) Prey and predator size relationship

We found that in cannibal events recorded in snakes, prey and predator size were positively correlated, in agreement with the theory of feeding strategies (Schoener, 1971), which predicts that an animal will invest most effort in consuming foods with a high energy return. It is also in line with predictions of foraging theory in snakes (Arnold, 1993) that larger predators will ingest larger prey unless smaller prey are widely available. The correlation between prey and predator size suggests that cannibalism in snakes may not be a maladaptive trait, but rather represents deliberate prey choice.

### (4) Mandibular morphotypes and evolution of cannibalism

Macrostomy (‘large mandible’) in snakes allows the consumption of larger prey, and is found in most extant snake species (Rieppel, 1988, 2012; Strong *et al*., 2021; Cundall & Irish, 2021). The free mandibular symphysis (symphyseal region with up to four elements) is a key element of this morphology, allowing a larger mouth gape and higher articulation, and therefore, better efficiency in ingestion and deglutition of prey (Rieppel, 1988; Cundall & Irish, 2008; Burbrink *et al*., 2020). The free mandibular symphysis is found in macrostomate snakes, with an intermediate condition represented by *Anilius scytale*, and a simple symphysis in microstomate scolecophidians (Esteves, 2010).

Due to the reduced flexibility and gape of the microstomate mandibular morphotypes (mandibular raking, single‐axle maxillary raking, and axle‐brace maxillary raking) it is unlikely that the paraphyletic scolecophidian snakes are physically able to swallow other snakes. An intriguing case is represented by the ‘snout‐shifting’ mandibular morphotype, as seen in *Anilius scytale*, which has a relatively reduced mouth gape and symphysis compared to other alethinophidians, but is able to prey upon other snakes. It is currently unclear whether the snout‐shifting morphology actually represents two distinct morphotypes or is a case of convergent evolution between Cylindrophiidae and Uropeltidae with Aniliidae. Furthermore, this morphology has not been evaluated for Tropidophiidae, the sister group of Aniliidae, which is known to be macrostomate and macrophagous (Rieppel, 2012; Ortega‐Andrade *et al*., 2022). The snout‐shifting morphotype arguably has significantly more mobility than scolecophidian counterparts, and therefore herein is provisionally considered as macrostomate until further evidence is able to test its origins under a phylogenetic framework. Cundall (1995) suggested that cylindrophiids with the snout‐shifting phenotype may have unilateral palatomaxillary arch movement and enhanced jaw mobility comparable with other macrostomate snakes.

Based on our ancestral character estimation we suggest that mandibular phenotypes have largely shaped the occurrence of cannibalism in extant snakes. The group of microstomy morphotypes, which is paraphyletic, appear to be physically precluded from handling conspecific prey; however, the ‘macrostomy morphotypes’ group, although likely monophyletic and biomechanically capable of conducting cannibalism, also has families in which this behaviour was not recorded. While this could be explained by the difficulty of recording feeding events in wild snakes, or the lack of general natural‐history data for several families, it should be noted that several clades have evolved highly specialized diets (e.g. dipsadine goo eaters; Oliveira *et al*., 2023), and may represent actual absences of this behaviour. Our continuous character mapping of ophiophagy for cannibal families revealed a higher number of records in Endoglyptodonta, which may suggest that the evolution of a venom‐delivery system could facilitate the predation of conspecifics. While we uncovered multiple cannibalism records in groups with species that have a specialized ophiophagous diet, a direct relationship between cannibal behaviour and the proportion of ophiophagy in their diet could not be verified in detail due to a lack of data on natural history for most species, and should be a focus of future studies.

## V. CONCLUSIONS

(1) Cannibalism is shown to be particularly prevalent in snake families with ophiophagous or generalist diets. Although half of recorded cannibalism events occurred in species with generalist diets, ophiophagous species also appear to have a higher occurrence of this behaviour, as seen in elapids. Cannibalism may also be associated with stress caused by lack of food.
(2) Maternal cannibalism was recorded most often in boid snakes, which may display maternal care. Conversely, occasional records of this behaviour in various snake taxa could be associated with stressors in captivity. Sexual cannibalism appears to be restricted to boids, and may constitute a foraging strategy. Combat‐dance cannibalism was recorded only in two elapid species which also have an ophiophagous diet. Cannibalism between offspring, although rarely recorded in snakes, may be related to sibling competition or resource availability.
(3) We found a positive correlation between prey and predator size in cannibalism events, in accordance with theoretical predictions of optimal feeding strategies.
(4) Cannibalism is restricted to taxa with a macrostomate mandibular morphology, i.e. to alethinophidians, and this behaviour may possibly be more prevalent in endoglyptodont advanced snakes.

## Supporting information


**Appendix S1.** Raw data set on snake cannibalism events, containing taxonomic information, diet, sex, size, age, event type, ingestion direction, country, year, and publication type.

**Appendix S2.** Data set on snake cannibalism events, containing log‐transformed values of total length (= snout–vent length + tail length) for predator and prey, mean percentage of cannibal species that are ophiophagous per family, and overview of ophiophagy records for cannibal species.
